# Global burden of inflammatory bowel disease in the elderly: trends from 1990 to 2021 and projections to 2051

**DOI:** 10.3389/fragi.2024.1479928

**Published:** 2024-10-24

**Authors:** Ying Liu, Ju Li, Guangxia Yang, Deqian Meng, Xianming Long, Kai Wang

**Affiliations:** ^1^ Department of Rheumatology and Immunology, The First People’s Hospital of Yancheng, The Fourth Affiliated Hospital of Nantong University, Yancheng, China; ^2^ Department of Rheumatology and Immunology, The Affiliated Huaian No.1 People’s Hospital of Nanjing Medical University, Huaian, China; ^3^ Department of Rheumatology, Affiliated Hospital of Jiangnan University, Wuxi, China; ^4^ Department of Rheumatology and Immunology, The First Affiliated Hospital of Soochow University, Suzhou, China

**Keywords:** inflammatory bowel disease, elderly, global burden, trends, projections

## Abstract

**Background:**

This study aims to analyze the historical trends of inflammatory bowel disease (IBD) burden in the elderly from 1990 to 2021 and forecast future trends up to 2051.

**Methods:**

Data from the Global Burden of Disease Study 2021 were utilized. Age-standardized rates (ASR) for incidence, prevalence, mortality, and disability-adjusted life years (DALYs) were calculated. Estimated annual percentage changes (EAPCs) were computed to quantify temporal trends. A Bayesian Age-Period-Cohort model was employed to project future trends.

**Results:**

From 1990 to 2021, the global number of elderly IBD increased from 573,500 to 1,278,190. The age-standardized incidence rate (ASIR) rose from 8.01 to 8.77 per 100,000, while the age-standardized prevalence rate (ASPR) slightly decreased from 118.14 to 117.29 per 100,000. Death number increased from 14,400 to 33,490, but the age-standardized mortality rate decreased from 3.21 to 2.84 per 100,000. DALYs increased from 324,100 to 683,750, with the age-standardized DALYs rate declining from 68.78 to 60.88 per 100,000. Significant geographical variations were observed, with high Socio-demographic Index regions showing the highest burden. Projections suggest that by 2051, elderly IBD prevalence number may reach 2,316,000, with ASIR and ASPR potentially rising after 2035 and 2042, respectively. Deaths and DALYs are projected to increase to 75,000 and 1,401,000 respectively, despite continued declines in ASRs.

**Conclusion:**

The absolute burden of IBD in the elderly population is projected to increase substantially by 2051, despite decreasing ASRs. These findings underscore the need for tailored healthcare strategies and resource allocation to address the growing challenge of elderly IBD globally.

## Introduction

Inflammatory bowel disease (IBD), primarily comprising Crohn’s disease and ulcerative colitis, is a group of chronic, relapsing inflammatory disorders affecting the gastrointestinal tract (Ramos and Papadakis, 2019). In recent decades, the global incidence and prevalence of IBD have risen significantly, emerging as a major public health concern (Ng et al., 2017). According to the latest Global Burden of Disease (GBD) Study, the number of IBD patients worldwide reached 6.8 million in 2017, an 83.8% increase from 1990 (Collaborators, 2020). This upward trend is not limited to Western developed countries but has also been observed in newly industrialized nations across Asia, Africa, and South America (Kaplan and Windsor, 2021). Environmental factors play a crucial role in IBD pathogenesis. Recent research suggests that industrialization, Western dietary patterns, increased antibiotic use, and environmental pollution may contribute to the rising incidence of IBD (Ananthakrishnan et al., 2018). Additionally, genetic factors and gut microbiota dysbiosis are considered important mechanisms in IBD development (Kostic et al., 2014).

Recent epidemiological studies have provided new insights into the burden of IBD in the elderly population. A nationwide population-based cohort study on elderly-onset IBD revealed that the incidence of IBD in individuals aged 60 and above has been steadily increasing over the past 2 decades (Singh et al., 2024). These findings highlight the growing importance of understanding and managing IBD in older adults. Furthermore, a Joinpoint regression analysis of global IBD burden trends from 1990 to 2019 uncovered divergent trends in different age cohorts. Notably, significant differences in IBD burden were found between the 10–24 and 50–69 age groups, suggesting age-specific factors influencing IBD epidemiology (Wang et al., 2024).

IBD not only impairs patients’ quality of life but also imposes a substantial economic burden on healthcare systems. A study of IBD patients in the United States revealed that their average annual medical expenditure in 2015–2016 was $22,987, more than triple that of non-IBD individuals (Park et al., 2020). Furthermore, IBD is associated with various complications and comorbidities, including depression, intestinal tumors, and cardiovascular diseases (Barberio et al., 2021; Rajamäki et al., 2021; Chen et al., 2022).

As the population ages, the proportion of elderly IBD patients continues to increase. The age-specific prevalence rate of IBD exhibited distinct peaks across different age groups. The highest prevalence was observed in the 60–64 years age bracket for one demographic group, while another group showed peak prevalence in the 70–74 years age range (Collaborators, 2020). A Canadian study showed that the percentage of IBD patients aged 60 and above rose from 18.9% in 1999 to 35.7% in 2018 (Coward et al., 2019). Elderly IBD patients face unique challenges, such as multiple comorbidities, drug interactions, and increased surgical risks (Taleban et al., 2015).

Despite significant advancements in IBD treatment over the past 2 decades, particularly with the introduction of biologics that have greatly improved patient outcomes, a considerable portion of patients still respond poorly to existing therapies or experience severe side effects (Neurath, 2017; Roda et al., 2020). Therefore, developing new treatment strategies, especially personalized approaches for elderly patients, remains a focus of current research (Danese et al., 2013).

Accurately predicting IBD trends over the next 30 years is crucial for effective prevention and management, given the rising global burden and complex factors involved. This study aims to analyze historical trends in the global burden of IBD and forecast developments over the next 3 decades, providing a scientific basis for global IBD control efforts.

## Methods

### Data source

This study utilized data from the GBD 2021, which includes IBD incidence, prevalence, deaths, and disability-adjusted life years (DALYs) across different age groups, genders, and geographical locations. Our analysis focused particularly on the elderly population (aged ≥60 years). Our selection of 60 years as the threshold for defining the elderly population aligns with the World Health Organization’s definition of older adults in developed countries and is consistent with the age limit used in the majority of IBD studies (Nørgård et al., 2024; Sivanathan et al., 2023).

### Data processing

We employed data from the GBD 2021 database, which encompasses various burden metrics for IBD across different demographic and geographic categories. Our analysis concentrated on the elderly population (aged ≥60 years). Age groups were divided into 18 intervals, with the 60+ population further stratified into “60–64”, “65–69”, “70–74”, “75–79”, “80–84”, “85–89”, “90–94”, and “95+ years” categories.

### Statistical analysis

Age-standardized rates (ASRs) for incidence, prevalence, deaths, and DALYs were calculated using the World Health Organization’s 2000–2025 World Standard Population as the reference. The calculation process was as follows: 1. Collect raw incidence numbers and population data for each region, year, and age group. 2. Calculate the specific incidence rate for each age group. 3. Calculate the weighted incidence rate for each age group (w[i] *r[i]). 4. Sum the weighted incidence rates to obtain the total ASR. The formula for ASR is: ASR = Σ(w[i] *r[i])/Σw[i]. Where w[i] is the weight for age group i in the standard population, and r[i] is the age-specific rate for age group i in the population of interest.

We calculated the estimated annual percentage change (EAPC) to quantify IBD burden trends from 1990 to 2021. The EAPC calculation process was as follows: 1. Take the natural logarithm of each year’s ASR, i.e., ln(ASR). 2. Establish the following linear regression model: ln(ASR) = β0 + β * (year - 1990) + ε. Where β0 is the intercept, β is the slope, and ε is the error term. 3. Calculate EAPC: EAPC = (e^β - 1) * 100%. Where e is the base of natural logarithms. The 95% uncertainty interval (UI) for EAPC was calculated as: 95% UI = [(e^(β - 1.96 * SE) - 1) * 100%, (e^(β + 1.96 * SE) - 1) * 100%]. Where SE is the standard error of β. A positive EAPC value indicates an upward trend in ASR during the study period, while a negative value suggests a downward trend. The EAPC value represents the average annual percentage change. If the 95% confidence interval of EAPC does not include 0, the trend is considered statistically significant.

An Age-Period-Cohort (APC) model, specifically the Bayesian age-period-cohort (BAPC) method, was employed to analyze and predict IBD trends in the elderly population. This model was chosen for its ability to simultaneously consider age, period, and cohort effects, making it particularly suitable for analyzing and predicting long-term trends in chronic diseases such as IBD (Riebler et al., 2016). The BAPC model was used to fit observed data from 1990 to 2021 and forecast trends from 2022 to 2051. Separate analyses were conducted for the total population, males, and females to compare gender differences.

All statistical analyses and visualizations were performed using R software (version 4.3.3). Key R packages utilized included ‘INLA’ for Bayesian inference, ‘BAPC’ for BAPC model, ‘reshape2’ for data reshaping, ‘tidyverse’ for data manipulation and visualization, and ‘ggplot2’ for creating graphics. A *p*-value <0.05 was considered statistically significant.

## Results

### Historical trends in global elderly IBD disease burden (1990–2021)

Global elderly IBD incidence more than doubled from 39,000 in 1990 to 95,240 in 2021, accounting for 25.4% of all IBD cases (Supplementary Tables 1, 2). The age-standardized incidence rate (ASIR) also showed an upward trend, increasing from 8.01 to 8.77. The prevalence similarly rose substantially, from 573,500 to 1,278,190, representing about 33.37% of all IBD prevalence in 2021 (Supplementary Table 2). However, the age-standardized prevalence rate (ASPR) slightly decreased from 118.14 to 117.29.

Gender differences were observed, with males showing slightly higher ASIR but females having a higher prevalence count, although ASPR was similar between genders (Supplementary Tables 3, 4). Geographically, high Socio-demographic Index (SDI) regions, particularly Australasia and high-income North America, exhibited the highest ASIR and ASPR. In contrast, East and Southeast Asian regions showed lower rates but with increasing trends.

The number of deaths increased from 14,400 to 33,490, accounting for 78.97% of all IBD deaths in 2021 (Supplementary Tables 5, 6). However, the age-standardized mortality rate (ASMR) showed a declining trend, decreasing from 3.21 to 2.84. DALYs rose from 324,100 to 683,750, representing 45.27% of all IBD DALYs in 2021 (Supplementary Tables 7, 8). The age-standardized DALYs rate (ASDR) also decreased from 68.78 to 60.88.

**FIGURE 1:**
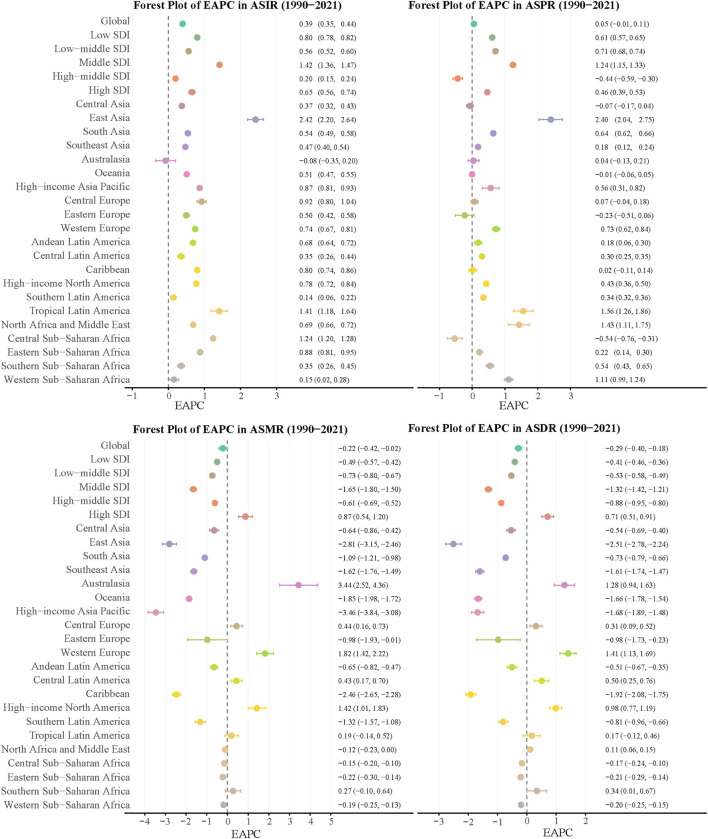
EAPCs of the ASRs for elderly IBD in Global and 26 regions. ASR, age-standardized rate; ASIR, age-standardized incidence rate; ASPR, age-standardized prevalence rate; ASMR, age-standardized mortality rate; DALYs, disability-adjusted life years; ASDR, age-standardized DALYs rate; EAPC, estimated annual percentage change; IBD, inflammatory bowel disease.

During the 1990–2021 period, global elderly IBD incidence and prevalence rates showed increasing trends, with EAPCs of 0.59 and 0.05 respectively (Figure 1). Conversely, mortality and DALYs rate exhibited declining trends, with EAPCs of −0.42 and −0.40 respectively. Regional disparities were evident, with East Asia showing the most significant increase in incidence rate (EAPC = 2.42), while high-income North America had the fastest growth in prevalence rate (EAPC = 0.43). Regarding mortality, Australasia demonstrated the most pronounced upward trend (EAPC = 3.44), while high-income Asia Pacific showed the most significant decline (EAPC = −3.46). In terms of disease burden, Australasia exhibited the largest increase (EAPC = 1.28), while high-income Asia Pacific again showed the most marked decrease (EAPC = −1.88).

### Age-stratified analysis of global elderly IBD burden (1990–2021)

**FIGURE 2:**
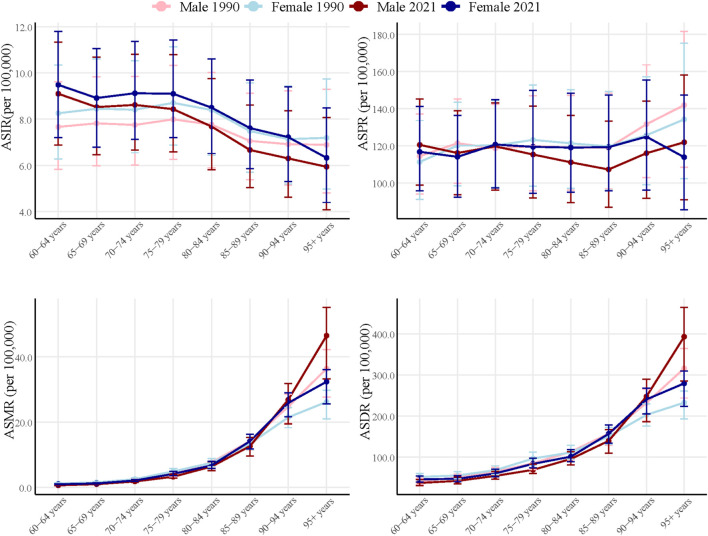
Global burden of Elderly IBD in 1990 and 2021 by sex and age group. ASIR, age-standardized incidence rate; ASPR, age-standardized prevalence rate; ASMR, age-standardized mortality rate; DALYs, disability-adjusted life years; ASDR, age-standardized DALYs rate; EAPC, estimated annual percentage change; IBD, inflammatory bowel disease.

The disease burden of elderly IBD exhibited significant changes from 1990 to 2021. ASIR increased across most age groups, with a more pronounced rise observed among females (Figure 2). While ASPR remained relatively stable over this 31-year period, a slight increase was noted in older age groups. ASMR and ASDR escalated sharply with age, particularly in individuals over 85 years old. Notably, both mortality and DALYs rates in 2021 were marginally higher than those in 1990 across most age groups.

**FIGURE 3:**
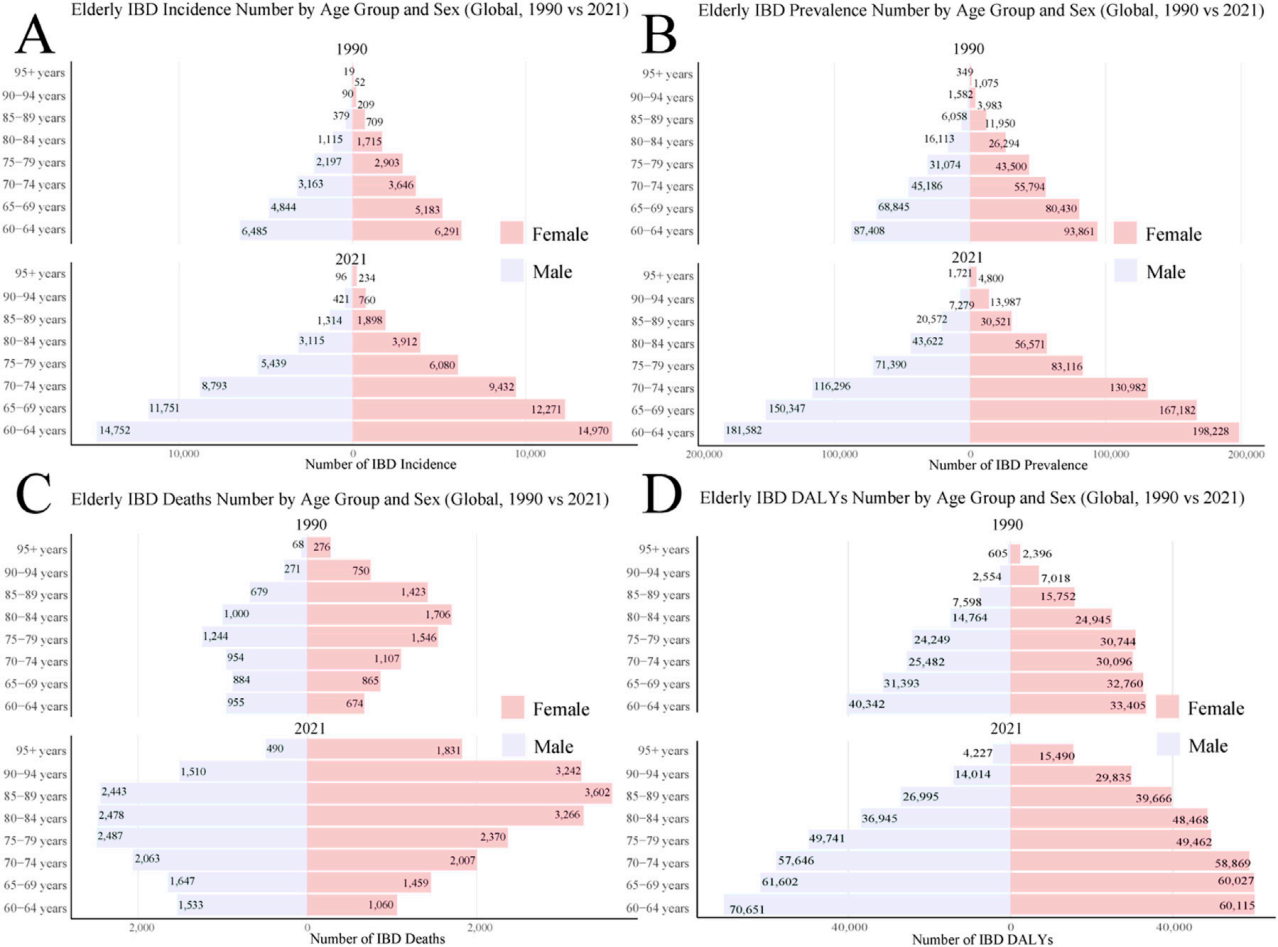
Global burden of Elderly IBD in 1990 and 2021 by sex and age group. **(A)** Elderly IBD incidence number; **(B)** Elderly IBD prevalence number; **(C)** Elderly IBD deaths number; **(D)** Elderly IBD DALYs number. DALYs, disability-adjusted life years; IBD, inflammatory bowel disease.

The number of incidence increased markedly in all age groups, with the most significant rise observed in the 60–74 years age bracket (Figures 3A, B). For instance, the number of incidence in the 60–64 years age group nearly doubled, from approximately 12,776 in 1990 to 29,722 in 2021. The growth in prevalence was even more substantial, with the same age group experiencing an increase from 181,269 in 1990 to 379,810 in 2021. Gender disparities were evident, with females showing slightly higher incidence and prevalence numbers than males in most age groups. Mortality figures also demonstrated an upward trend, particularly pronounced in the older age groups (85 years and above) (Figures 3C, D). A striking example is the more than six-fold increase in deaths among those aged 95 and over, rising from 344 in 1990 to 2,321 in 2021.

### Geographic distribution of global elderly IBD disease burden (2021 status)

**FIGURE 4:**
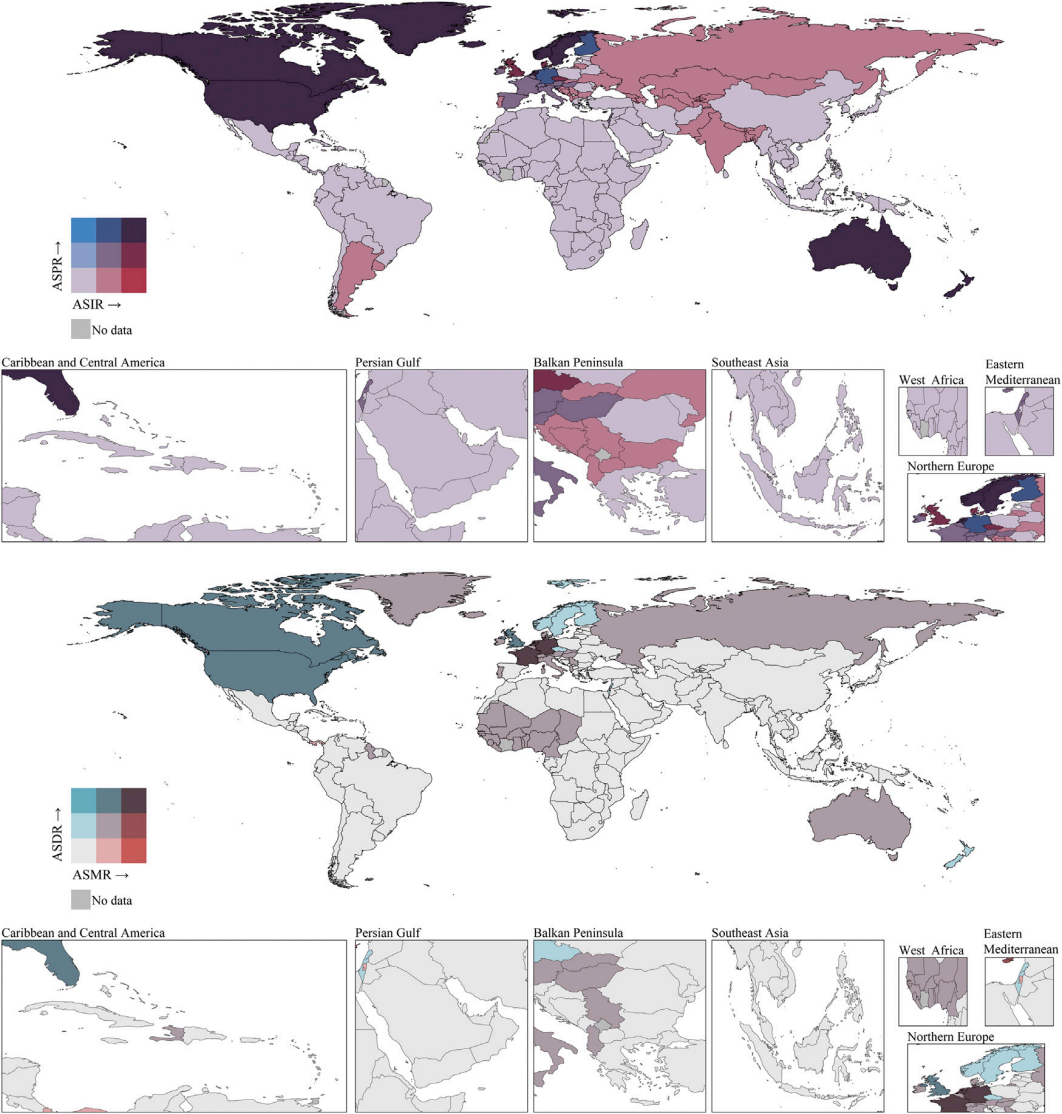
ASIRs, ASPRs, ASMRs, and ASDRs of elderly IBD in 2021 by country (or territory). IBD, inflammatory bowel disease; ASIR, age standardized incidence rate; ASPR, age-standardized prevalence rate; ASMR, age-standardized mortality rate; ASDR, age-standardized disability-adjusted life-years rate.

The incidence and prevalence of elderly IBD showed distinct geographical variations (Figure 4A). Developed countries and regions, such as North America (particularly the United States and Canada), Western Europe, Australasia, and New Zealand, exhibited the highest incidence and prevalence rates. In contrast, most regions in Africa, the Middle East, and South America showed lower rates. Significant disparities were also observed within Asia, with developed East Asian countries like Japan and South Korea showing markedly higher rates than Southeast and South Asian regions.

Similar geographical disparities were observed in mortality rates and disease burden (Figure 4B). North America (especially the US and Canada), parts of Western Europe, and Australasia showed higher ASMR and ASDR. Most Asian, African, and South American countries had relatively lower ASMR and ASDR. Notably, some West African countries and North African regions displayed moderate levels of mortality and disease burden. Within Europe, clear differences were observed, with Northern and Western European countries generally showing higher ASMR and ASDR than Eastern and Southern European countries.

### Relationship between socioeconomic development and elderly IBD disease burden

**FIGURE 5:**
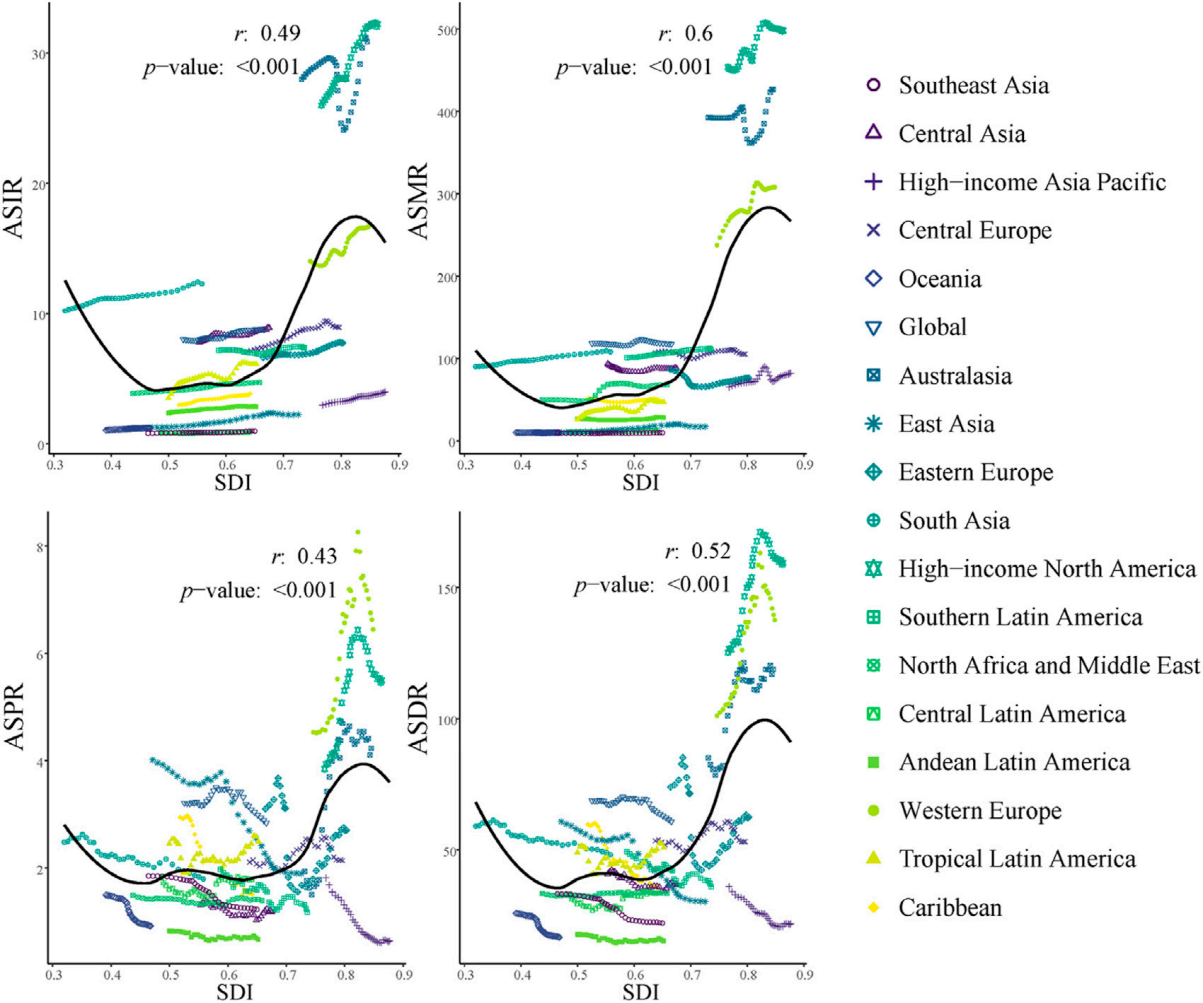
ASRs for elderly IBD in different GBD regions by SDI, 1990–2021. IBD, inflammatory bowel disease; ASIR, age standardized incidence rate; ASPR, age-standardized prevalence rate; ASMR, age-standardized mortality rate; ASDR, age-standardized disability-adjusted life-years rate.

At the regional level (Figure 5), ASIR, ASPR, ASMR), and ASDR all showed positive correlations with SDI (*r* = 0.49, 0.6, 0.43, and 0.52 respectively; all *p* < 0.001). Significant disparities were observed among regions, with high-income areas such as North America and Western Europe exhibiting generally higher indicator values compared to low-SDI regions like Central and Southeast Asia. Eastern Europe and South Asia demonstrated a sharp increase in indicators at higher SDI levels, reflecting unique challenges faced during rapid economic development.

At the national level (Supplementary Figure 1), ASIR and ASPR exhibited even stronger positive correlations with SDI (*r* = 0.5 and 0.55 respectively, *p* < 0.001). However, the correlation between ASMR and SDI was weak and insignificant (*r* = 0.06, *p* = 0.44), potentially reflecting the mitigating effect of improved healthcare on mortality rates despite increased incidence. ASDR showed a weak to moderate positive correlation with SDI (*r* = 0.2, *p* < 0.01). Notably, substantial variations in indicators were observed even among high-SDI countries such as New Zealand, Canada, and the Netherlands.

### Future trends prediction for global elderly IBD disease burden (2022–2051)

**FIGURE 6:**
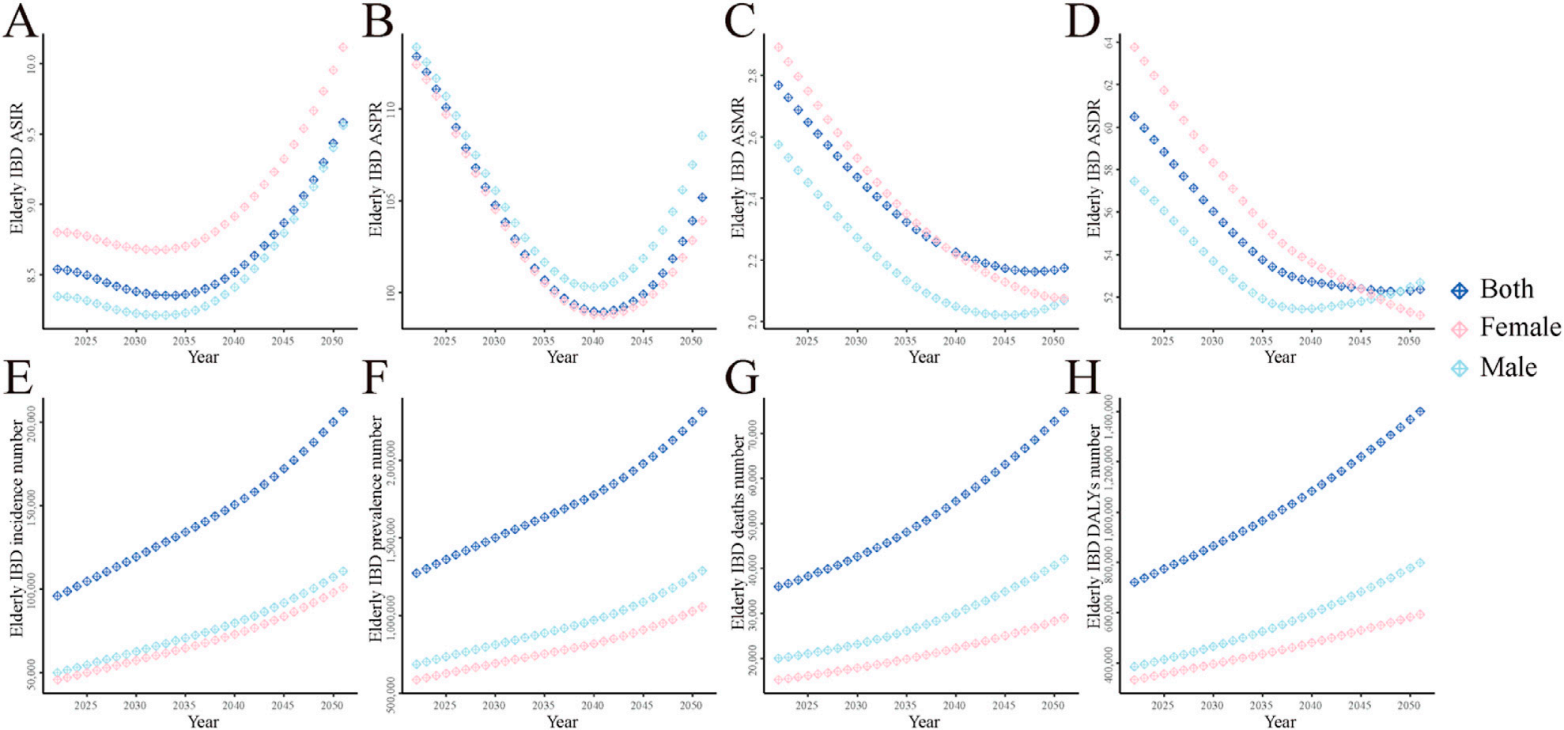
The global disease burden of elderly IBD predicted by BAPC model from 2022 to 2051. **(A)** ASIR; **(B)** ASPR; **(C)** ASMR; **(D)** ASDR; **(E)** Incidence number; **(F)** Prevalence number; **(G)** Deaths number; **(H)** DALYs. ASIR, age-standardized incidence rate; ASPR, age-standardized prevalence rate; ASMR, age-standardized mortality rate; DALYs, disability-adjusted life years; ASDR, age-standardized DALYs rate; EAPC, estimated annual percentage change; IBD, inflammatory bowel disease.

Predictions based on the BAPC model indicated that ASIR and ASPR would initially decrease and then increase (Figures 6A, B). ASIR is expected to start rising in 2035, reaching approximately 9.58 by 2051, while ASPR is predicted to begin increasing in 2042, reaching about 105.18 by 2051. In contrast, ASMR and ASDR are projected to continuously decline, reaching approximately 2.17 and 52.36 respectively by 2051 (Figures 6C, D).

The predictions suggest that females will have higher ASIR and ASPR than males, while males will show slightly higher ASMR and ASDR (Figures 6A–D). In absolute number, incidence, prevalence, deaths, and DALYs for elderly IBD are all projected to increase continuously, reaching approximately 206,000, 2,316,000, 75,000, and 1,401,000 respectively by 2051 (Figures 6E–H).

## Discussion

This study comprehensively analyzed global trends in the disease burden of elderly IBD from 1990 to 2021 and predicted future trends for the next 30 years using the BAPC model. Our findings reveal that while age-standardized incidence and prevalence rates for elderly IBD have remained relatively stable, absolute number have shown significant growth, closely correlating with global aging trends (Ng et al., 2017).

Elderly IBD burden generally increases with socioeconomic development, as indicated by the SDI (Windsor and Kaplan, 2019). However, this relationship is not linear. The global trend line demonstrates an acceleration of disease burden at medium SDI levels, highlighting the complex interplay between societal development and health outcomes (Ng et al., 2019). This non-linear pattern suggests that as countries transition through different stages of development, they may face varying challenges in managing elderly IBD (Kaplan and Ng, 2017).

The observed variability among countries with similar SDI levels indicates that factors beyond socioeconomic development significantly influence the IBD burden in the elderly population (Ananthakrishnan, 2015). These factors may include genetic predisposition, environmental exposures, dietary habits, and differences in healthcare systems and policies (Molodecky et al., 2012). Such diversity underscores the need for tailored approaches in addressing elderly IBD across different global contexts (Bernstein, 2017).

These findings have important implications for future healthcare planning and policy development. They provide a foundation for predicting IBD trends in the elderly population over the next 3 decades, allowing for more accurate forecasting of healthcare needs (Coward et al., 2019). Moreover, they emphasize the importance of developing targeted prevention and management strategies that consider not only the socioeconomic status of a region or country but also its unique genetic, environmental, and healthcare characteristics (Ng et al., 2017). This nuanced understanding is crucial for effectively addressing the growing challenge of elderly IBD in an increasingly aging global population (Beard et al., 2016).

Our findings align with recent studies on the global burden of IBD. An analysis of GBD 2021 data reported a consistent increase in IBD burden worldwide from 1990 to 2021, with notable variations across regions and age groups (Lin et al., 2024). These results corroborate our observations on the rising prevalence of IBD in the elderly population.

The COVID-19 pandemic has also impacted IBD epidemiology. A comparison of two nationwide cohorts examining IBD epidemiology during the pandemic found changes in diagnosis patterns and healthcare utilization (Atia et al., 2024). While our study does not specifically address the pandemic’s impact, these findings underscore the need for continued monitoring of IBD trends in the context of global health crises.

Additionally, a recent study highlighted the prevalence and challenges of polypharmacy in older adults with IBD (Kochar et al., 2024). This aspect is particularly relevant to our findings on the increasing burden of IBD in the elderly, as it emphasizes the complexity of managing IBD in this population and the potential for increased healthcare utilization and costs.

Our research indicates a rising proportion of elderly IBD patients among total IBD number, consistent with previous studies. For instance, Coward et al. reported an increase in the proportion of IBD patients aged 60 and above from 18.9% in 1999 to 35.7% in 2018 (Coward et al., 2019). This trend not only reflects the impact of population aging but may also be attributed to extended survival of IBD patients. Advancements in treatment modalities, particularly the widespread use of biologics, have significantly improved patient outcomes, leading to more patients entering elderly stages (Peyrin-Biroulet et al., 2015).

The age-stratified analysis of global elderly IBD burden from 1990 to 2021 reveals important patterns and trends across different age groups. Notably, incidence rates increased across most age groups, with a more pronounced rise among females, suggesting potential gender-specific risk factors or diagnostic patterns (Bernstein et al., 2006). While prevalence rates remained relatively stable over the 31-year period, slight increases were observed in older age groups, possibly reflecting improved survival rates and disease management (Charpentier et al., 2014). The sharp escalation of mortality and DALYs rates with age, particularly in individuals over 85 years old, underscores the cumulative impact of disease duration and the challenges of managing IBD in the very elderly (Ananthakrishnan et al., 2009). The substantial growth in absolute numbers of incident and prevalent cases, especially in the 60–74 years age bracket, highlights the increasing burden on healthcare systems and the need for targeted interventions for this age group (Coward et al., 2019). The more than six-fold increase in deaths among those aged 95 and over is particularly concerning and may reflect both population aging and the complexities of IBD management in the oldest old (Nguyen et al., 2015). These findings emphasize the need for age-specific management strategies, increased focus on early intervention in the younger elderly, and further research into the unique characteristics and needs of elderly-onset IBD (Taleban et al., 2015). Furthermore, the observed gender disparities in disease burden call for more attention to potential biological and societal factors influencing IBD development and progression in elderly males and females (Shah et al., 2018).

Regarding geographical disparities, we found that high SDI regions, especially North America and Western Europe, exhibit the highest incidence and prevalence rates, aligning with the global epidemiological study in 2017 (Ng et al., 2017). Notably, we observed the most significant growth in incidence rates in East Asia (EAPC = 2.42), potentially linked to rapid industrialization and Westernization in the region, leading to changes in environmental factors and lifestyles (Kaplan, 2015).

In terms of mortality and disease burden, despite increases in absolute number, age-standardized rates show declining trends. This reflects improvements in IBD diagnosis and treatment, particularly in developed countries (Zhao et al., 2021). However, we noted an upward trend in mortality rates in Australasia (EAPC = 3.44), a phenomenon warranting further investigation and possibly related to local healthcare policies, population structure, or environmental factors.

Our 30-year forecast suggests that incidence and prevalence rates of elderly IBD may rise again after 2035. This trend could stem from continued population aging and further advancements in diagnostic techniques. Concurrently, mortality rates and disease burden are projected to continue decreasing, reflecting ongoing progress in medical technology and management strategies (Aniwan et al., 2017).

Regarding gender differences, we predict higher incidence and prevalence rates for females, while males show slightly higher mortality rates and disease burden. These disparities may be attributed to physiological factors, environmental exposures, and healthcare-seeking behaviors (Greuter et al., 2020). Future research should delve deeper into the specific causes of these differences to develop more targeted prevention and treatment strategies.

While this study provides important insights into the global burden of IBD in the elderly population, several limitations must be acknowledged. Firstly, our reliance on the GBD 2021 database may have resulted in incomplete data collection in certain regions, particularly in low- and middle-income countries. Some countries and regions have estimates with wide confidence intervals, possibly due to limitations in data collection and insufficient sample sizes. This suggests that caution is needed when interpreting results for these areas. Secondly, variations in IBD definitions and diagnostic criteria across countries may have affected the accuracy of cross-regional comparisons. Thirdly, our choice of 60 years as the threshold for defining the elderly population may not be universally applicable, especially considering the global increase in life expectancy. Although this selection aligns with the World Health Organization’s definition of older adults and is consistent with many IBD studies, it may overestimate the IBD burden in the “young-old” (60–74 years) population. In some developed countries, 65 years might be a more appropriate threshold. This choice may not adequately reflect the nuanced differences in IBD burden across various age subgroups within the elderly population. Future research should consider further stratification of the elderly population or adjusting age thresholds based on regional contexts to provide more precise analyses. Fourthly, while we employed the advanced BAPC model for projections, all predictive models have inherent uncertainties. Long-term projections, in particular, may be influenced by unforeseen factors such as advances in medical technology, environmental changes, or global events. Fifthly, our study did not differentiate between IBD subtypes or disease severity. Additionally, we were unable to fully account for other important factors that may influence IBD diagnosis, treatment, and prognosis, such as socioeconomic status, comorbidities, polypharmacy, lifestyle, and environmental factors. Lastly, the use of aggregated data precluded individual-level analyses, limiting our ability to conduct in-depth investigations of risk factors and prognostic indicators. These limitations provide direction for future research, particularly in improving data collection, refining disease classification, considering additional influential factors, and more precisely defining and stratifying the elderly population.

Based on our findings, we recommend: 1) Enhancing early screening and diagnosis for elderly IBD patients, especially in regions with rapidly growing incidence rates; 2) Developing personalized treatment plans for elderly patients, considering their comorbidities and medication profiles; 3) Intensifying research on the relationship between environmental factors and IBD onset to inform prevention strategies; 4) Improving long-term follow-up and management systems for elderly IBD patients to reduce complications and enhance quality of life.

In conclusion, this study provides a comprehensive analysis of the global burden of IBD in the elderly population from 1990 to 2021 and projects trends to 2051. Our findings reveal a significant increase in elderly IBD cases, with notable geographical variations and gender disparities. Despite decreasing age-standardized rates, absolute numbers of cases, deaths, and DALYs are projected to rise. These results underscore the growing challenge of elderly IBD and the need for targeted healthcare strategies. This study offers valuable insights for policymakers and healthcare providers to allocate resources effectively and develop age-specific management strategies. Future research should focus on understanding the unique aspects of elderly-onset IBD and improving diagnosis and treatment for this growing patient population.

## Data Availability

Publicly available datasets were analyzed in this study. This data can be found here: https://vizhub.healthdata.org/gbd-results/.
