# Supplementary material for: Global burden of inflammatory bowel disease in the elderly: trends from 1990 to 2021 and projections to 2051
Source: Front Aging. 2024 Oct 24;5:1479928. doi: 10.3389/fragi.2024.1479928 (PMC11540814; doi:10.3389/fragi.2024.1479928)
Supplement: Supplementary file 1 [file DataSheet1.docx]

**
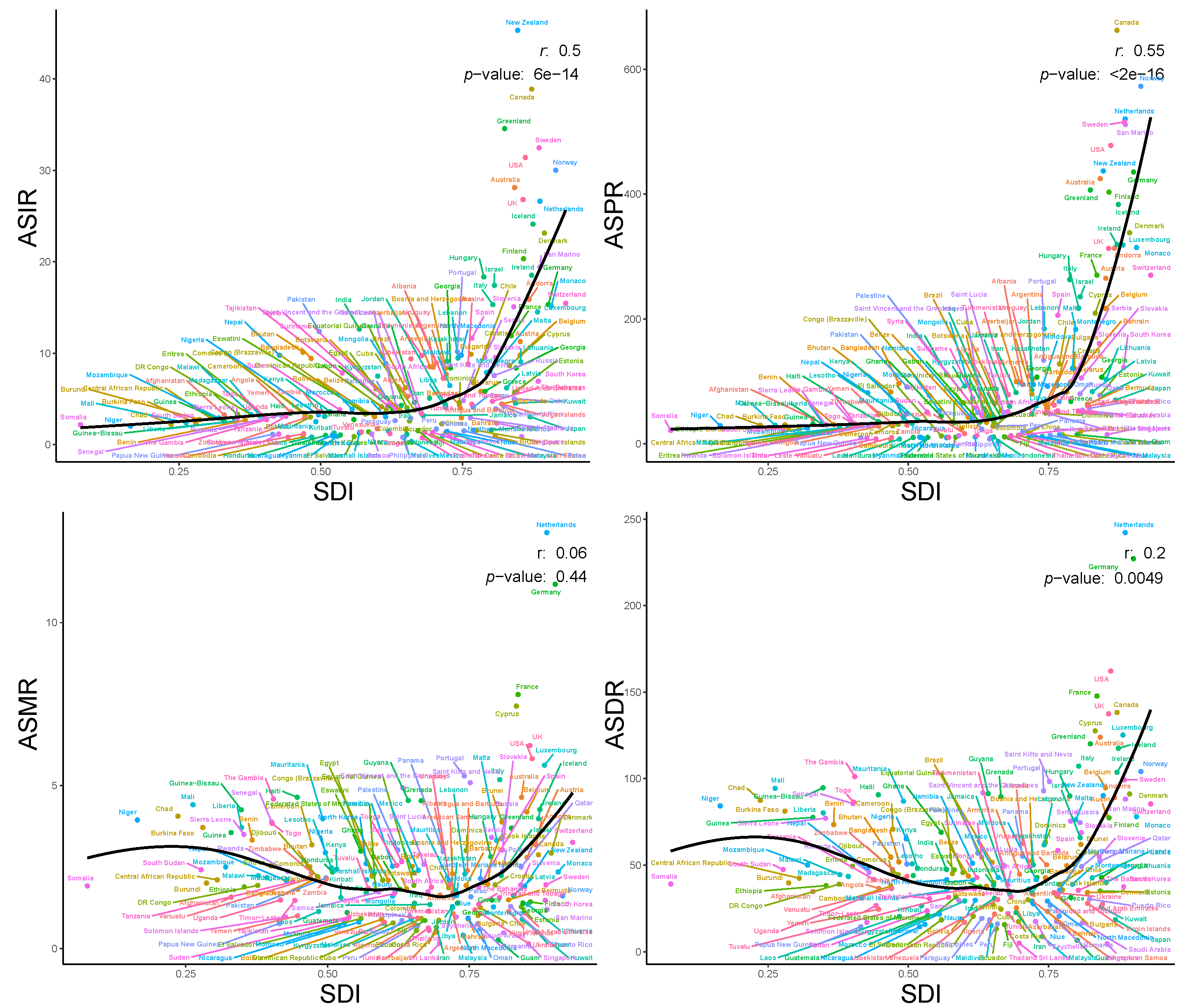
**

**Supplementary Fiqure 1.** ASRs for elderly IBD in 204 countries and territories by SDI, 1990-2021.

IBD, inflammatory bowel disease; ASIR, age standardized incidence rate; ASPR, age-standardized prevalence rate; ASMR, age-standardized mortality rate; ASDR, age-standardized disability-adjusted life-years rate.

## **Supplementary** Table 1. Elderly IBD incidence and ASIR in 1990 and 2021.

|  | **1990** | | **2021** | |
| --- | --- | --- | --- | --- |
| **Characteristics** | **Incidence cases** | **ASIR** | **Incidence cases** | **ASIR** |
|  | **No. ×10^3^ (95% UI)** | **(95% UI)** | **No. ×10^3^ (95% UI)** | **(95% UI)** |
| Overall | 39 (29.94-49.3) | 8.01 (6.15-10.13) | 95.24 (72.78-119.43) | 8.77 (6.7-10.99) |
| Sex |  |  |  |  |
| Male | 18.29 (14.05-23) | 8.35 (6.42-10.52) | 45.68 (35.08-56.94) | 9.04 (6.95-11.27) |
| Female | 20.71 (15.88-26.28) | 7.73 (5.93-9.81) | 49.56 (37.76-62.38) | 8.54 (6.51-10.74) |
| Socio-demographic index |  |  |  |  |
| Low | 1.3 (0.96-1.69) | 4.97 (3.67-6.47) | 3.63 (2.71-4.69) | 6.31 (4.72-8.16) |
| Low-middle | 5.02 (3.7-6.54) | 7.07 (5.23-9.22) | 14.67 (10.89-19.02) | 8.49 (6.3-11) |
| Middle | 3.35 (2.46-4.37) | 2.73 (2.01-3.57) | 13.88 (10.28-17.99) | 4.17 (3.09-5.4) |
| Middle-high | 6.85 (5.13-8.85) | 5.4 (4.05-6.98) | 14.78 (10.96-19.14) | 5.79 (4.29-7.49) |
| High | 22.44 (17.55-27.89) | 15.71 (12.29-19.49) | 48.2 (37.64-59.08) | 18.26 (14.27-22.32) |
| Region |  |  |  |  |
| Central Asia | 0.44 (0.33-0.58) | 7.77 (5.69-10.2) | 0.9 (0.65-1.18) | 8.89 (6.49-11.66) |
| East Asia | 1.34 (0.99-1.75) | 1.27 (0.94-1.66) | 6.25 (4.64-8.1) | 2.25 (1.67-2.91) |
| South Asia | 6.72 (4.97-8.74) | 10.21 (7.56-13.29) | 22.07 (16.46-28.52) | 12.27 (9.15-15.86) |
| Southeast Asia | 0.24 (0.17-0.32) | 0.81 (0.58-1.08) | 0.79 (0.57-1.04) | 0.97 (0.71-1.29) |
| Australasia | 0.86 (0.66-1.09) | 27.98 (21.5-35.3) | 2.11 (1.61-2.67) | 30.88 (23.53-39.08) |
| Oceania | 0 (0-0) | 1.03 (0.74-1.38) | 0.01 (0.01-0.01) | 1.23 (0.88-1.64) |
| High-income Asia Pacific | 0.76 (0.56-0.99) | 2.97 (2.18-3.89) | 2.14 (1.58-2.79) | 3.98 (2.93-5.17) |
| Central Europe | 1.4 (1.05-1.79) | 7.07 (5.32-9.08) | 2.61 (1.95-3.34) | 8.92 (6.65-11.4) |
| Eastern Europe | 2.46 (1.78-3.25) | 6.64 (4.82-8.78) | 3.71 (2.7-4.88) | 7.74 (5.64-10.16) |
| Western Europe | 10.47 (8.21-13.03) | 14.02 (11-17.4) | 18.86 (14.44-23.71) | 16.71 (12.8-20.92) |
| Andean Latin America | 0.06 (0.04-0.07) | 2.34 (1.67-3.14) | 0.2 (0.15-0.27) | 2.84 (2.05-3.74) |
| Central Latin America | 0.08 (0.06-0.11) | 0.87 (0.62-1.16) | 0.29 (0.2-0.39) | 0.92 (0.64-1.26) |
| Caribbean | 0.1 (0.07-0.13) | 3 (2.16-3.94) | 0.25 (0.19-0.33) | 3.84 (2.82-5.02) |
| High-income North America | 11.84 (9.25-14.65) | 25.98 (20.32-32.06) | 28.05 (22.2-34.13) | 32.21 (25.51-39.12) |
| Southern Latin America | 0.42 (0.3-0.57) | 7.17 (5.02-9.73) | 0.82 (0.58-1.1) | 7.39 (5.24-9.95) |
| Tropical Latin America | 0.38 (0.28-0.49) | 3.51 (2.63-4.54) | 1.97 (1.46-2.56) | 6.13 (4.55-7.95) |
| North Africa and Middle East | 0.77 (0.56-1.01) | 3.86 (2.82-5.06) | 2.5 (1.81-3.3) | 4.71 (3.42-6.21) |
| Central Sub-IBDaran Africa | 0.06 (0.05-0.08) | 2.37 (1.75-3.11) | 0.2 (0.15-0.26) | 3.36 (2.5-4.36) |
| Eastern Sub-IBDaran Africa | 0.22 (0.16-0.28) | 2.55 (1.9-3.32) | 0.6 (0.45-0.78) | 3.21 (2.4-4.15) |
| Southern Sub-IBDaran Africa | 0.1 (0.08-0.13) | 3.21 (2.4-4.11) | 0.24 (0.18-0.31) | 3.43 (2.55-4.44) |
| Western Sub-IBDaran Africa | 0.27 (0.2-0.36) | 2.66 (1.96-3.46) | 0.64 (0.48-0.83) | 2.94 (2.18-3.81) |

IBD, inflammatory bowel disease; ASIR, age standardized incidence rate; UI, uncertainty interval; EAPC, estimated annual percentage change.

**Supplementary Table 2.** IBD incidence and ASIR in 1990 and 2021, and its temporal trends from 1990 to 2021.

|  | **1990** | | **2021** | | **1990-2021** |
| --- | --- | --- | --- | --- | --- |
| **Characteristics** | **Incidence cases** | **ASIR** | **Incidence cases** | **ASIR** | **EAPC** |
|  | **No. ×10^3^ (95% UI)** | **(95% UI)** | **No. ×10^3^ (95% UI)** | **No. (95% UI)** | **(95% UI)** |
| Overall | 199.24 (174.58-232.68) | 4.2 (3.7-4.9) | 375.14 (327.69-436.93) | 4.4 (3.9-5.2) | 0.29 (0.2-0.38) |
| Socio-demographic index |  |  |  |  |  |
| Low | 8.37 (7.16-10.14) | 2.5 (2.2-3.1) | 23.06 (19.69-27.96) | 3 (2.6-3.6) | 0.57 (0.54-0.61) |
| Low-middle | 30.78 (26.41-37.16) | 3.6 (3.2-4.3) | 75.38 (64.73-91.31) | 4.3 (3.7-5.1) | 0.55 (0.49-0.62) |
| Middle | 22.85 (19.54-27.84) | 1.6 (1.4-1.9) | 64.89 (56.02-78.45) | 2.4 (2-2.9) | 1.54 (1.38-1.7) |
| Middle-high | 32.33 (28.39-37.89) | 3 (2.6-3.5) | 53.49 (46.61-63.3) | 3.3 (2.8-3.9) | 0.48 (0.33-0.64) |
| High | 104.71 (93.32-120.55) | 10.6 (9.5-12.3) | 158.05 (139.74-180.17) | 11.6 (10.1-13.4) | 0.33 (0.21-0.46) |
| Region |  |  |  |  |  |
| Central Asia | 2.52 (2.18-3.07) | 4.4 (3.8-5.3) | 5.02 (4.32-6.1) | 5.2 (4.5-6.2) | 0.5 (0.47-0.53) |
| East Asia | 8.63 (7.28-10.59) | 0.7 (0.6-0.9) | 25.53 (22.11-30.53) | 1.4 (1.2-1.7) | 2.88 (2.37-3.4) |
| South Asia | 40.23 (34.46-48.84) | 5 (4.3-6) | 106.22 (91.6-128.25) | 6 (5.2-7.2) | 0.66 (0.58-0.74) |
| Southeast Asia | 2.27 (1.92-2.8) | 0.6 (0.5-0.7) | 5.04 (4.27-6.18) | 0.7 (0.6-0.8) | 0.51 (0.47-0.55) |
| Australasia | 4 (3.42-4.74) | 18.1 (15.5-21.4) | 7.31 (6.37-8.47) | 19.7 (17.1-23.2) | 0.48 (0.31-0.65) |
| Oceania | 0.03 (0.03-0.04) | 0.6 (0.6-0.8) | 0.09 (0.08-0.11) | 0.8 (0.6-0.9) | 0.46 (0.41-0.5) |
| High-income Asia Pacific | 4.18 (3.63-4.95) | 2.2 (1.9-2.6) | 6.53 (5.69-7.66) | 2.8 (2.4-3.3) | 1.1 (0.63-1.57) |
| Central Europe | 7.32 (6.41-8.62) | 5.4 (4.7-6.3) | 9.3 (8.13-10.75) | 6.5 (5.6-7.7) | 0.79 (0.6-0.97) |
| Eastern Europe | 8.9 (7.71-10.72) | 3.4 (3-4.1) | 11.23 (9.68-13.55) | 4.1 (3.6-4.9) | 0.57 (0.53-0.62) |
| Western Europe | 50.6 (45.35-57.68) | 11.7 (10.4-13.4) | 66.44 (58.28-76.43) | 12.7 (11.1-15) | 0.32 (0.17-0.47) |
| Andean Latin America | 0.4 (0.34-0.48) | 1.4 (1.2-1.6) | 1.05 (0.9-1.3) | 1.6 (1.4-2) | 0.56 (0.5-0.62) |
| Central Latin America | 0.74 (0.63-0.9) | 0.6 (0.5-0.7) | 1.5 (1.26-1.85) | 0.6 (0.5-0.7) | 0.15 (0.03-0.27) |
| Caribbean | 0.73 (0.62-0.88) | 2.3 (1.9-2.7) | 1.38 (1.18-1.66) | 2.7 (2.3-3.3) | 0.55 (0.49-0.61) |
| High-income North America | 52.44 (46.63-59.95) | 16.8 (14.9-19.2) | 84.76 (75.25-95.39) | 18.5 (16.3-21.2) | 0.37 (0.26-0.48) |
| Southern Latin America | 2.15 (1.85-2.63) | 4.5 (3.9-5.5) | 3.61 (3.13-4.38) | 4.7 (4.1-5.8) | 0.19 (0.13-0.24) |
| Tropical Latin America | 2.36 (2.03-2.88) | 1.9 (1.6-2.3) | 7.62 (6.55-9.29) | 2.9 (2.5-3.6) | 1.03 (0.55-1.51) |
| North Africa and Middle East | 7.18 (6.13-8.7) | 2.7 (2.3-3.3) | 19.66 (16.59-24.19) | 3.2 (2.7-3.9) | 0.56 (0.51-0.6) |
| Central Sub-Saharan Africa | 0.51 (0.43-0.62) | 1.4 (1.2-1.6) | 1.75 (1.49-2.16) | 1.8 (1.6-2.2) | 1.02 (0.99-1.04) |
| Eastern Sub-Saharan Africa | 1.64 (1.41-2) | 1.4 (1.2-1.6) | 4.9 (4.18-6) | 1.7 (1.5-2) | 0.8 (0.74-0.86) |
| Southern Sub-Saharan Africa | 0.63 (0.54-0.76) | 1.6 (1.4-2) | 1.28 (1.1-1.55) | 1.7 (1.5-2) | 0.26 (0.17-0.35) |
| Western Sub-Saharan Africa | 1.78 (1.53-2.15) | 1.4 (1.2-1.6) | 4.93 (4.24-5.97) | 1.5 (1.3-1.8) | 0.11 (0-0.22) |

IBD, inflammatory bowel disease; ASIR, age standardized incidence rate; UI, uncertainty interval; EAPC, estimated annual percentage change; UI, uncertainty interval.

**Supplementary Table 3.** IBD prevalence and ASPR in 1990 and 2021, and its temporal trends from 1990 to 2021.

|  | **1990** | | **2021** | | **1990-2021** |
| --- | --- | --- | --- | --- | --- |
| **Characteristics** | **Prevalence cases** | **ASPR** | **Prevalence cases** | **ASPR** | **EAPC** |
|  | **No. ×10^3^ (95% UI)** | **(95% UI)** | **No. ×10^3^ (95% UI)** | **No. (95% UI)** | **(95% UI)** |
| Overall | 2170.24 (1892.4-2522.56) | 48 (41.9-55.8) | 3830.12 (3312.83-4511.55) | 44.9 (38.8-52.9) | -0.13 (-0.25-0) |
| Socio-demographic index |  |  |  |  |  |
| Low | 64.11 (53.9-77.41) | 20.7 (17.5-25) | 166.79 (140.41-203.99) | 22.8 (19.4-27.6) | 0.41 (0.36-0.46) |
| Low-middle | 228 (192.79-273.56) | 28 (23.7-33.5) | 561.44 (475.6-680.84) | 32.5 (27.5-39.1) | 0.66 (0.58-0.73) |
| Middle | 194.22 (163.8-235.11) | 14 (11.9-16.9) | 544.52 (460.46-659.9) | 19.6 (16.6-23.8) | 1.34 (1.19-1.5) |
| Middle-high | 374.81 (323.92-442.31) | 35.3 (30.6-41.6) | 541.77 (462.61-647.49) | 31.6 (27.1-37.9) | -0.15 (-0.31-0.02) |
| High | 1306.71 (1155.69-1487.81) | 129.5 (114.4-147.6) | 2012.48 (1755.44-2316.53) | 132.8 (115-154.3) | 0.08 (-0.1-0.26) |
| Region |  |  |  |  |  |
| Central Asia | 25.3 (21.39-30.48) | 46.2 (39-55.9) | 44.28 (37.28-53.73) | 45.6 (38.6-55) | 0.04 (-0.02-0.11) |
| East Asia | 64.81 (54.67-78.25) | 5.6 (4.8-6.7) | 172.2 (145.04-206.86) | 9.1 (7.7-10.9) | 2.49 (1.87-3.11) |
| South Asia | 301.26 (253.27-365.31) | 38.9 (33-46.9) | 802.54 (676.7-975.73) | 46.2 (39.2-55.7) | 0.73 (0.63-0.83) |
| Southeast Asia | 20.95 (17.48-25.17) | 5.6 (4.7-6.6) | 42.51 (35.57-51.6) | 5.7 (4.8-6.9) | 0.17 (0.12-0.21) |
| Australasia | 42.86 (36.69-51.49) | 192 (164.1-230.8) | 80.7 (69.82-95) | 203.3 (174.7-240.9) | 0.56 (0.3-0.82) |
| Oceania | 0.28 (0.23-0.34) | 5.8 (4.8-7.2) | 0.64 (0.53-0.79) | 5.6 (4.6-6.8) | -0.18 (-0.23--0.14) |
| High-income Asia Pacific | 59.82 (51.06-71.29) | 30 (25.6-35.9) | 103.66 (88.51-124.31) | 37.3 (31.6-45) | 0.9 (0.44-1.36) |
| Central Europe | 91.96 (79.26-107.72) | 65.8 (56.7-77) | 102.43 (88.12-120.33) | 67.1 (57.4-78.8) | 0.21 (0.03-0.38) |
| Eastern Europe | 97.79 (82.91-118.52) | 37 (31.5-44.8) | 101.04 (85.76-121.7) | 35.4 (30-42.6) | -0.03 (-0.2-0.14) |
| Western Europe | 651.53 (571.49-737.27) | 143 (125.8-162.6) | 929.73 (806-1081.44) | 156.5 (134.5-181.9) | 0.18 (-0.01-0.38) |
| Andean Latin America | 3.64 (3.08-4.38) | 13.2 (11.2-15.8) | 8.92 (7.56-10.77) | 13.6 (11.6-16.4) | 0.08 (-0.02-0.19) |
| Central Latin America | 6.62 (5.51-8.08) | 5.6 (4.7-6.7) | 14.7 (12.24-17.65) | 5.6 (4.6-6.7) | 0.09 (-0.01-0.19) |
| Caribbean | 8.41 (7.02-10.03) | 27.9 (23.3-33.3) | 14.19 (11.97-16.92) | 27.5 (23.2-32.8) | 0.01 (-0.04-0.05) |
| High-income North America | 648.15 (574.64-742.48) | 203.4 (180.3-233.5) | 997.97 (872.99-1147.78) | 198.4 (172.5-231.7) | -0.02 (-0.18-0.13) |
| Southern Latin America | 23.18 (19.57-28.04) | 48.9 (41.3-59.4) | 41.02 (34.73-50.2) | 52.4 (44.3-64.4) | 0.24 (0.22-0.25) |
| Tropical Latin America | 15.85 (13.44-19.02) | 12.8 (11-15.2) | 52.83 (44.7-63.51) | 20.4 (17.3-24.4) | 1.14 (0.67-1.61) |
| North Africa and Middle East | 70.5 (59.01-84.3) | 29.2 (24.6-34.8) | 215.4 (179.47-259.04) | 35.7 (29.9-42.9) | 1 (0.78-1.22) |
| Central Sub-Saharan Africa | 4.42 (3.73-5.29) | 13.2 (11.2-15.9) | 11.35 (9.61-13.81) | 12.5 (10.6-15) | -0.33 (-0.5--0.16) |
| Eastern Sub-Saharan Africa | 12.51 (10.51-15.08) | 11.3 (9.5-13.6) | 34.88 (29.44-42) | 12.9 (10.9-15.4) | 0.26 (0.19-0.33) |
| Southern Sub-Saharan Africa | 5.11 (4.34-6.12) | 14.4 (12.3-17.2) | 12.19 (10.35-14.77) | 17.3 (14.8-20.7) | 0.43 (0.37-0.49) |
| Western Sub-Saharan Africa | 15.3 (12.87-18.5) | 12.8 (10.8-15.5) | 46.95 (39.67-56.94) | 15.8 (13.5-19.2) | 0.76 (0.71-0.82) |

IBD, inflammatory bowel disease; ASPR, age-standardized prevalence rate; UI, uncertainty interval; EAPC, estimated annual percentage change; UI, uncertainty interval.

## **Supplementary** Table 4. Elderly IBD prevalence and ASPR in 1990 and 2021.

|  | **1990** | | **2021** | |
| --- | --- | --- | --- | --- |
| **Characteristics** | **Prevalence cases** | **ASPR** | **Prevalence cases** | **ASPR** |
|  | **No. ×10^3^ (95% UI)** | **(95% UI)** | **No. ×10^3^ (95% UI)** | **(95% UI)** |
| Overall | 573.5 (467.92-692.48) | 118.14 (96.33-142.74) | 1278.19 (1033.03-1547.28) | 117.29 (94.84-141.95) |
| Sex |  |  |  |  |
| Male | 256.62 (208.79-309.95) | 117.94 (95.82-142.75) | 592.81 (478.72-719.29) | 117.49 (94.85-142.67) |
| Female | 316.89 (258.09-382.85) | 118.41 (96.45-143.02) | 685.39 (553.51-828.24) | 117.33 (94.83-141.65) |
| Socio-demographic index |  |  |  |  |
| Low | 12.2 (9.38-15.47) | 46.63 (35.86-59.28) | 31.52 (24.27-40.15) | 54.81 (42.2-69.96) |
| Low-middle | 43.37 (33.5-55.26) | 61.02 (47.09-77.9) | 126.57 (98.2-160.65) | 73.16 (56.76-92.95) |
| Middle | 34.78 (27.03-44.08) | 28.34 (22-35.99) | 134.93 (104.95-171.44) | 40.52 (31.52-51.5) |
| Middle-high | 101.46 (80.93-125.35) | 80.11 (63.85-99.03) | 175 (136.76-219.54) | 68.39 (53.48-85.75) |
| High | 381.13 (313.85-454.74) | 266.77 (220.01-317.72) | 809.24 (661.72-962.91) | 303.88 (249.09-360.58) |
| Region |  |  |  |  |
| Central Asia | 5.39 (4.12-6.89) | 93.55 (71.35-119.71) | 8.94 (6.92-11.44) | 87.79 (67.71-112.48) |
| East Asia | 10.79 (8.22-13.88) | 10.27 (7.82-13.24) | 48.16 (37.13-61.38) | 17.28 (13.33-22.02) |
| South Asia | 59.45 (46.02-75.52) | 90.05 (69.62-114.69) | 194.74 (150.64-247.73) | 108.17 (83.68-137.74) |
| Southeast Asia | 2.82 (2.13-3.67) | 9.46 (7.15-12.34) | 8 (6.08-10.35) | 9.85 (7.47-12.77) |
| Australasia | 12.04 (9.57-14.81) | 392.57 (312.55-482.46) | 28.97 (23.09-35.49) | 426.63 (341.28-520.65) |
| Oceania | 0.03 (0.03-0.04) | 10.16 (7.53-13.38) | 0.08 (0.06-0.11) | 10.18 (7.58-13.41) |
| High-income Asia Pacific | 16.56 (13.27-20.53) | 65.13 (52.2-80.72) | 46.06 (36.68-57.22) | 82.05 (65.84-101.51) |
| Central Europe | 21.15 (16.72-26.17) | 107.23 (84.68-132.66) | 30.8 (23.85-38.69) | 105.3 (81.71-132.09) |
| Eastern Europe | 31.97 (24.69-40.64) | 86.57 (66.82-110.09) | 36.63 (28.17-47.34) | 76.07 (58.5-98.31) |
| Western Europe | 175.91 (144.04-211.71) | 237.34 (194.78-284.77) | 347.62 (280.91-421.87) | 307.91 (249.71-371.97) |
| Andean Latin America | 0.63 (0.47-0.82) | 26.38 (19.87-34.37) | 2.02 (1.53-2.6) | 28.12 (21.31-36.3) |
| Central Latin America | 1.06 (0.82-1.37) | 11 (8.44-14.22) | 3.68 (2.79-4.75) | 11.89 (9.01-15.33) |
| Caribbean | 1.65 (1.28-2.09) | 51.63 (40.06-65.45) | 3.32 (2.55-4.25) | 50.01 (38.53-63.96) |
| High-income North America | 208.01 (172.57-245.32) | 453.63 (377.12-533.84) | 436.49 (357.3-515.81) | 497.88 (408.34-587.55) |
| Southern Latin America | 5.98 (4.55-7.59) | 101.18 (77.01-128.56) | 12.22 (9.34-15.67) | 110.78 (84.96-141.69) |
| Tropical Latin America | 2.77 (2.14-3.51) | 25.71 (19.79-32.55) | 15.08 (11.36-19.42) | 46.75 (35.23-60.17) |
| North Africa and Middle East | 10.01 (7.67-12.68) | 49.71 (38.02-63.12) | 36.29 (28.08-45.84) | 67.96 (52.51-86.07) |
| Central Sub-IBDaran Africa | 0.76 (0.58-0.98) | 29.53 (22.57-37.87) | 1.57 (1.19-2.02) | 26.28 (19.98-33.74) |
| Eastern Sub-IBDaran Africa | 2.23 (1.72-2.83) | 26.06 (20.11-33.08) | 5.55 (4.28-7.03) | 29.67 (22.85-37.65) |
| Southern Sub-IBDaran Africa | 1.22 (0.96-1.52) | 38.39 (30.03-47.96) | 3.31 (2.63-4.1) | 48.36 (38.33-59.9) |
| Western Sub-IBDaran Africa | 3.08 (2.38-3.9) | 30 (23.11-38.04) | 8.68 (6.78-10.84) | 39.98 (31.14-50) |

IBD, inflammatory bowel disease; ASPR, age-standardized prevalence rate; UI, uncertainty interval; EAPC, estimated annual percentage change.

## **Supplementary** Table 5. Elderly IBD death and ASMR in 1990 and 2021.

|  | **1990** | | **2021** | |
| --- | --- | --- | --- | --- |
| **Characteristics** | **Death cases** | **ASMR** | **Death cases** | **ASMR** |
|  | **No. ×10^3^ (95% UI)** | **(95% UI)** | **No. ×10^3^ (95% UI)** | **(95% UI)** |
| Overall | 14.4 (12.03-16.14) | 3.21 (2.68-3.6) | 33.49 (28.31-38.23) | 2.84 (2.42-3.24) |
| Sex |  |  |  |  |
| Male | 6.06 (5.12-7.07) | 3.29 (2.78-3.83) | 14.65 (12.66-17) | 3.02 (2.61-3.51) |
| Female | 8.35 (6.56-9.71) | 3.11 (2.44-3.62) | 18.84 (14.99-22.99) | 2.67 (2.16-3.28) |
| Socio-demographic index |  |  |  |  |
| Low | 0.59 (0.41-0.79) | 2.59 (1.76-3.51) | 1.13 (0.82-1.51) | 2.2 (1.6-2.95) |
| Low-middle | 1.45 (0.97-1.91) | 2.35 (1.55-3.13) | 3.01 (2.3-4.02) | 1.89 (1.43-2.51) |
| Middle | 2.8 (1.68-3.64) | 2.8 (1.66-3.67) | 5.73 (4.42-6.95) | 1.79 (1.38-2.18) |
| Middle-high | 3.1 (2.74-3.49) | 2.67 (2.35-3.01) | 6.23 (5.23-7.67) | 2.22 (1.88-2.74) |
| High | 6.46 (5.76-6.92) | 4.02 (3.59-4.29) | 17.35 (14.4-19.19) | 4.6 (3.93-5.03) |
| Region |  |  |  |  |
| Central Asia | 0.07 (0.06-0.08) | 1.31 (1.16-1.5) | 0.11 (0.09-0.13) | 1.19 (1.01-1.4) |
| East Asia | 2.88 (1.71-3.79) | 4.02 (2.34-5.3) | 5.08 (3.83-7.24) | 1.9 (1.43-2.71) |
| South Asia | 1.38 (0.89-1.88) | 2.48 (1.56-3.42) | 2.95 (2.09-4.14) | 1.8 (1.27-2.52) |
| Southeast Asia | 0.47 (0.22-0.74) | 1.85 (0.84-2.94) | 0.87 (0.57-1.1) | 1.23 (0.81-1.55) |
| Australasia | 0.06 (0.05-0.07) | 1.94 (1.64-2.24) | 0.42 (0.33-0.5) | 4.23 (3.36-5) |
| Oceania | 0 (0-0.01) | 1.5 (0.73-2.55) | 0.01 (0-0.01) | 0.93 (0.57-1.46) |
| High-income Asia Pacific | 0.45 (0.34-0.56) | 1.81 (1.35-2.25) | 0.61 (0.45-0.85) | 0.65 (0.5-0.88) |
| Central Europe | 0.4 (0.37-0.44) | 2.11 (1.93-2.35) | 0.72 (0.63-0.8) | 2.14 (1.89-2.39) |
| Eastern Europe | 1.12 (1.01-1.25) | 3.1 (2.81-3.47) | 1.35 (1.21-1.47) | 2.7 (2.44-2.95) |
| Western Europe | 4.01 (3.56-4.32) | 4.53 (4.03-4.87) | 12 (9.79-13.43) | 6.44 (5.41-7.15) |
| Andean Latin America | 0.02 (0.01-0.03) | 0.84 (0.56-1.2) | 0.05 (0.04-0.08) | 0.68 (0.47-0.98) |
| Central Latin America | 0.17 (0.16-0.18) | 1.83 (1.7-1.95) | 0.58 (0.51-0.64) | 1.79 (1.58-1.99) |
| Caribbean | 0.09 (0.08-0.11) | 2.95 (2.53-3.47) | 0.11 (0.09-0.14) | 1.51 (1.19-1.95) |
| High-income North America | 2.06 (1.81-2.21) | 3.84 (3.41-4.11) | 5.75 (4.86-6.25) | 5.5 (4.76-5.93) |
| Southern Latin America | 0.12 (0.1-0.13) | 1.98 (1.72-2.24) | 0.15 (0.13-0.17) | 1.19 (1.02-1.35) |
| Tropical Latin America | 0.25 (0.22-0.27) | 2.45 (2.22-2.65) | 0.84 (0.73-0.92) | 2.49 (2.17-2.71) |
| North Africa and Middle East | 0.25 (0.18-0.36) | 1.5 (1.04-2.17) | 0.66 (0.49-0.91) | 1.4 (1.03-1.93) |
| Central Sub-IBDaran Africa | 0.04 (0.02-0.06) | 2.03 (1.2-3.22) | 0.09 (0.05-0.15) | 1.93 (1.14-3.08) |
| Eastern Sub-IBDaran Africa | 0.16 (0.1-0.22) | 2.2 (1.38-3.19) | 0.33 (0.2-0.48) | 2.08 (1.25-3) |
| Southern Sub-IBDaran Africa | 0.05 (0.03-0.08) | 1.78 (1-2.71) | 0.11 (0.08-0.15) | 1.85 (1.32-2.41) |
| Western Sub-IBDaran Africa | 0.35 (0.23-0.53) | 3.56 (2.31-5.36) | 0.68 (0.48-0.91) | 3.31 (2.35-4.36) |

IBD, inflammatory bowel disease; ASMR, age-standardized mortality rate; UI, uncertainty interval; EAPC, estimated annual percentage change.

**Supplementary Table 6.** IBD death and ASMR in 1990 and 2021, and its temporal trends from 1990 to 2021.

|  | **1990** | | **2021** | | **1990-2021** |
| --- | --- | --- | --- | --- | --- |
| **Characteristics** | **Deaths** | **ASMR** | **Deaths** | **ASMR** | **EAPC** |
|  | **No. ×10^3^ (95% UI)** | **(95% UI)** | **No. ×10^3^ (95% UI)** | **No. (95% UI)** | **(95% UI)** |
| Overall | 21.42 (18.42-23.61) | 0.6 (0.5-0.7) | 42.42 (37.54-46.5) | 0.5 (0.5-0.6) | -0.31 (-0.48--0.14) |
| Socio-demographic index |  |  |  |  |  |
| Low | 1.3 (0.92-1.61) | 0.5 (0.4-0.7) | 2.74 (1.91-3.42) | 0.5 (0.3-0.6) | -0.42 (-0.49--0.35) |
| Low-middle | 2.88 (2.1-3.51) | 0.5 (0.3-0.6) | 5.21 (4.31-6.49) | 0.4 (0.3-0.5) | -0.77 (-0.82--0.71) |
| Middle | 4.9 (3.46-5.92) | 0.5 (0.3-0.6) | 7.91 (6.47-9.1) | 0.3 (0.3-0.4) | -1.67 (-1.8--1.53) |
| Middle-high | 4.69 (4.31-5.21) | 0.5 (0.5-0.6) | 7.44 (6.48-8.79) | 0.4 (0.3-0.5) | -0.99 (-1.09--0.89) |
| High | 7.62 (7.02-8.01) | 0.7 (0.7-0.7) | 19.08 (16.2-20.66) | 0.8 (0.7-0.9) | 0.84 (0.52-1.17) |
| Region |  |  |  |  |  |
| Central Asia | 0.2 (0.18-0.22) | 0.4 (0.3-0.4) | 0.26 (0.22-0.3) | 0.3 (0.3-0.4) | -1.03 (-1.26--0.8) |
| East Asia | 4.59 (3.15-5.76) | 0.7 (0.5-0.9) | 5.97 (4.7-7.91) | 0.3 (0.3-0.4) | -3.01 (-3.35--2.66) |
| South Asia | 2.74 (1.9-3.53) | 0.5 (0.3-0.6) | 4.72 (3.51-6.29) | 0.3 (0.3-0.5) | -1.21 (-1.31--1.1) |
| Southeast Asia | 0.77 (0.42-1.08) | 0.3 (0.2-0.5) | 1.24 (0.89-1.47) | 0.2 (0.2-0.3) | -1.61 (-1.75--1.48) |
| Australasia | 0.07 (0.07-0.08) | 0.3 (0.3-0.4) | 0.45 (0.37-0.5) | 0.7 (0.6-0.8) | 3.54 (2.62-4.46) |
| Oceania | 0.01 (0.01-0.02) | 0.4 (0.2-0.6) | 0.02 (0.01-0.03) | 0.3 (0.2-0.4) | -1.88 (-2.02--1.73) |
| High-income Asia Pacific | 0.59 (0.47-0.69) | 0.3 (0.3-0.4) | 0.67 (0.52-0.89) | 0.1 (0.1-0.2) | -3.42 (-3.77--3.07) |
| Central Europe | 0.59 (0.56-0.65) | 0.4 (0.4-0.5) | 0.86 (0.79-0.95) | 0.4 (0.4-0.4) | 0.11 (-0.16-0.38) |
| Eastern Europe | 1.8 (1.65-1.98) | 0.7 (0.6-0.7) | 1.83 (1.67-1.97) | 0.5 (0.5-0.6) | -1.43 (-2.13--0.71) |
| Western Europe | 4.58 (4.21-4.8) | 0.8 (0.7-0.8) | 12.79 (10.65-13.95) | 1.1 (1-1.2) | 1.71 (1.31-2.12) |
| Andean Latin America | 0.06 (0.04-0.08) | 0.2 (0.2-0.3) | 0.09 (0.07-0.11) | 0.1 (0.1-0.2) | -1.37 (-1.61--1.13) |
| Central Latin America | 0.31 (0.3-0.32) | 0.4 (0.3-0.4) | 0.89 (0.8-0.98) | 0.4 (0.3-0.4) | 0.5 (0.22-0.78) |
| Caribbean | 0.16 (0.14-0.18) | 0.6 (0.5-0.7) | 0.19 (0.15-0.23) | 0.4 (0.3-0.4) | -2.08 (-2.27--1.89) |
| High-income North America | 2.42 (2.18-2.54) | 0.7 (0.6-0.7) | 6.6 (5.76-7.05) | 1 (0.9-1) | 1.49 (1.1-1.88) |
| Southern Latin America | 0.17 (0.16-0.18) | 0.4 (0.4-0.4) | 0.19 (0.18-0.21) | 0.2 (0.2-0.2) | -1.4 (-1.64--1.16) |
| Tropical Latin America | 0.49 (0.47-0.51) | 0.5 (0.5-0.5) | 1.3 (1.2-1.37) | 0.5 (0.5-0.5) | 0.11 (-0.2-0.42) |
| North Africa and Middle East | 0.5 (0.38-0.71) | 0.3 (0.2-0.4) | 1.06 (0.86-1.39) | 0.3 (0.2-0.3) | -0.36 (-0.47--0.24) |
| Central Sub-Saharan Africa | 0.09 (0.06-0.12) | 0.4 (0.3-0.6) | 0.22 (0.15-0.32) | 0.4 (0.3-0.5) | -0.09 (-0.15--0.03) |
| Eastern Sub-Saharan Africa | 0.32 (0.23-0.42) | 0.4 (0.3-0.6) | 0.74 (0.49-1) | 0.4 (0.3-0.6) | -0.16 (-0.24--0.08) |
| Southern Sub-Saharan Africa | 0.11 (0.08-0.14) | 0.4 (0.2-0.5) | 0.21 (0.16-0.25) | 0.4 (0.3-0.5) | 0.19 (-0.2-0.58) |
| Western Sub-Saharan Africa | 0.85 (0.58-1.12) | 0.8 (0.5-1.1) | 2.14 (1.34-2.91) | 0.8 (0.5-1) | 0.03 (-0.01-0.06) |

IBD, inflammatory bowel disease; ASPR, age-standardized prevalence rate; UI, uncertainty interval; EAPC, estimated annual percentage change; UI, uncertainty interval.

## **Supplementary** Table 7. Elderly IBD DALYs and ASDR in 1990 and 2021.

|  | **1990** | | **2021** | |
| --- | --- | --- | --- | --- |
| **Characteristics** | **DALYs** | **ASDR** | **DALYs** | **ASDR** |
|  | **No. ×10^3^ (95% UI)** | **(95% UI)** | **No. ×10^3^ (95% UI)** | **(95% UI)** |
| Overall | 324.1 (273.96-371.03) | 68.78 (58.07-78.68) | 683.75 (582.62-788.42) | 60.88 (52.02-70.25) |
| Sex |  |  |  |  |
| Male | 146.99 (125.07-171.21) | 71.68 (61.01-83.36) | 321.82 (277.78-374.08) | 64.84 (55.97-75.33) |
| Female | 177.12 (144.46-205.9) | 65.96 (53.76-76.71) | 361.93 (298.13-434.87) | 57.31 (47.52-69) |
| Socio-demographic index |  |  |  |  |
| Low | 14.03 (10.47-18.18) | 56.52 (41.6-73.76) | 27.61 (21.07-35.38) | 49.92 (38.02-64.2) |
| Low-middle | 35.07 (25.63-44.13) | 52.17 (37.74-66.09) | 74.88 (58.9-96.01) | 44.62 (35.07-57.26) |
| Middle | 55.82 (36.02-70.57) | 50.56 (32.1-64.44) | 115.05 (92.04-137.14) | 35.25 (28.16-42.05) |
| Middle-high | 69.92 (61.54-79.17) | 57.08 (50.12-64.64) | 118.93 (100.74-142.76) | 44.64 (37.9-53.52) |
| High | 148.95 (127.61-172.24) | 99.2 (84.68-115.32) | 346.74 (288.63-401.06) | 113.51 (95.16-132.3) |
| Region |  |  |  |  |
| Central Asia | 2.32 (1.95-2.74) | 40.83 (34.46-48.3) | 3.62 (3-4.37) | 36.67 (30.45-44.05) |
| East Asia | 51.43 (31.63-66.86) | 60.85 (36.68-79.44) | 83.27 (63.74-114.23) | 30.41 (23.27-41.83) |
| South Asia | 36.63 (26.53-46.95) | 59.08 (42.17-76.32) | 84.13 (62.99-111.35) | 48.15 (36.04-63.81) |
| Southeast Asia | 9.03 (4.48-13.74) | 33.3 (16.26-51.05) | 16.57 (11.52-20.5) | 22.28 (15.38-27.63) |
| Australasia | 2.65 (2-3.44) | 85.08 (63.75-110.62) | 9.28 (7.16-11.5) | 118.81 (91.17-149.1) |
| Oceania | 0.07 (0.03-0.12) | 25.92 (13.14-43.17) | 0.12 (0.07-0.18) | 16.81 (10.76-25.85) |
| High-income Asia Pacific | 9.09 (7.16-11.2) | 36.01 (28.35-44.34) | 14.49 (11.07-19.06) | 21.97 (16.8-28.61) |
| Central Europe | 10.4 (9.1-11.91) | 53.52 (46.85-61.28) | 16.37 (14.16-18.93) | 53.14 (45.89-61.64) |
| Eastern Europe | 27.12 (24.16-30.63) | 73.99 (65.94-83.52) | 30.37 (27.09-33.68) | 62.55 (55.87-69.36) |
| Western Europe | 81.91 (70.72-93.72) | 101.16 (86.86-116.71) | 198.45 (163.98-227.76) | 137.63 (115-159.56) |
| Andean Latin America | 0.43 (0.31-0.58) | 18.07 (13.02-24.56) | 1.15 (0.84-1.57) | 15.58 (11.42-21.13) |
| Central Latin America | 3.14 (2.93-3.34) | 33.15 (30.95-35.27) | 10.54 (9.34-11.74) | 33.67 (29.89-37.48) |
| Caribbean | 1.92 (1.66-2.26) | 59.76 (51.38-70.11) | 2.43 (1.95-3.09) | 35.56 (28.35-45.24) |
| High-income North America | 60.15 (49.71-71.89) | 125.25 (103.1-150.78) | 147.19 (122.99-172.47) | 159.48 (133.63-187.64) |
| Southern Latin America | 2.92 (2.47-3.46) | 49.38 (41.79-58.43) | 4.16 (3.36-5.15) | 36 (29.01-44.81) |
| Tropical Latin America | 5.16 (4.74-5.57) | 49.03 (44.89-53) | 16.69 (14.73-18.43) | 50.91 (45.11-56.16) |
| North Africa and Middle East | 6.21 (4.72-8.37) | 33.4 (25.17-45.28) | 16.97 (13.22-22) | 33.42 (25.98-43.4) |
| Central Sub-IBDaran Africa | 0.93 (0.6-1.41) | 41.05 (26.28-62.21) | 2.09 (1.32-3.23) | 38.92 (24.55-59.99) |
| Eastern Sub-IBDaran Africa | 3.47 (2.41-4.66) | 43.77 (29.79-59.96) | 7.34 (4.8-10.08) | 41.87 (27.21-57.84) |
| Southern Sub-IBDaran Africa | 1.15 (0.72-1.66) | 37.15 (23.14-53.59) | 2.66 (2.05-3.35) | 40.19 (30.94-50.7) |
| Western Sub-IBDaran Africa | 7.98 (5.28-11.84) | 78.37 (52-116.08) | 15.84 (11.41-20.69) | 72.98 (52.74-95.05) |

IBD, inflammatory bowel disease; DALYs, disability-adjusted life years; ASDR, age standardized DALYs rate; UI, uncertainty interval; EAPC, estimated annual percentage change.

**Supplementary Table 8.** IBD DALYs and ASDR in 1990 and 2021, and its temporal trends from 1990 to 2021.

|  | **1990** | | **2021** | | **1990-2021** |  |
| --- | --- | --- | --- | --- | --- | --- |
| **Characteristics** | **DALYs** | **ASDR** | **DALYs** | **ASDR** | **EAPC** |  |
|  | **No. ×10^3^ (95% UI)** | **(95% UI)** | **No. ×10^3^ (95% UI)** | **No. (95% UI)** | **(95% UI)** |  |
| Overall | 948.86 (808.1-1096.72) | 21.5 (18.5-24.8) | 1510.78 (1308.51-1750.36) | 18.1 (15.7-20.9) | -0.52 (-0.6--0.43) |  |
| Socio-demographic index |  |  |  |  |  |  |
| Low | 61.7 (45.77-79.57) | 19.2 (14.3-23.6) | 138.08 (98.06-170.35) | 18 (13.3-21.8) | -0.25 (-0.3--0.2) |  |
| Low-middle | 141.89 (112.36-174.78) | 17.6 (13.7-21.3) | 257.85 (210.81-312.04) | 15.4 (12.6-18.7) | -0.45 (-0.49--0.41) |  |
| Middle | 205.68 (155.79-244.16) | 15.7 (11.7-18.4) | 285.3 (240.2-329.98) | 10.9 (9.2-12.6) | -1.23 (-1.28--1.17) |  |
| Middle-high | 194.74 (169.86-224.66) | 19.3 (16.9-22.3) | 229.28 (197.04-267.17) | 13.1 (11.2-15.2) | -1.4 (-1.49--1.31) |  |
| High | 343.88 (283.24-418.65) | 33.7 (27.6-41.2) | 599.04 (501.8-711.06) | 35 (28.3-43) | 0.31 (0.1-0.51) |  |
| Region |  |  |  |  |  |  |
| Central Asia | 13.07 (10.96-15.11) | 21.4 (18.4-24.7) | 17.04 (14.25-20.61) | 17.9 (15-21.6) | -0.83 (-0.97--0.68) |  |
| East Asia | 167.5 (120.09-212.7) | 18.3 (12.8-23) | 143.57 (116.16-180.09) | 7.8 (6.3-9.6) | -2.89 (-3.16--2.62) |  |
| South Asia | 146.28 (114.03-186.34) | 19.5 (15-24.3) | 269.36 (211.77-341.92) | 16.1 (12.7-20.4) | -0.64 (-0.7--0.58) |  |
| Southeast Asia | 28.47 (17.97-36.11) | 8.8 (5.3-11.8) | 40.67 (30.95-47.97) | 6.1 (4.6-7.2) | -1.42 (-1.53--1.31) |  |
| Australasia | 7.71 (5.51-10.32) | 34.5 (24.5-46.1) | 18.18 (14.28-22.72) | 41.7 (31.9-53.8) | 1.08 (0.75-1.41) |  |
| Oceania | 0.55 (0.3-0.78) | 13.2 (7.2-18.9) | 0.88 (0.63-1.24) | 8.5 (6.1-11.6) | -1.74 (-1.89--1.6) |  |
| High-income Asia Pacific | 22.94 (18.87-27.27) | 12.1 (10-14.3) | 26.08 (20.51-32.42) | 8.4 (6.4-10.7) | -1 (-1.16--0.85) |  |
| Central Europe | 30.68 (25.95-36.47) | 22.2 (18.8-26.4) | 33.74 (28.35-40.51) | 20 (16.4-24.2) | -0.08 (-0.28-0.11) |  |
| Eastern Europe | 67.53 (59.87-76.77) | 25.5 (22.6-29) | 60.78 (54.39-68.51) | 20.1 (17.8-22.7) | -1.41 (-1.86--0.95) |  |
| Western Europe | 180.62 (149.91-216.86) | 37.6 (30.7-45.7) | 318.24 (270.22-372.59) | 42.9 (34.9-52.3) | 0.66 (0.41-0.91) |  |
| Andean Latin America | 3.71 (2.62-5.14) | 10.6 (8-13.7) | 4.06 (3.24-5.11) | 6.3 (5.1-8) | -1.73 (-2.01--1.44) |  |
| Central Latin America | 11.77 (11.22-12.38) | 10.4 (10-10.8) | 26.77 (24.12-29.56) | 10.4 (9.4-11.5) | 0.49 (0.23-0.76) |  |
| Caribbean | 6.47 (5.49-7.61) | 21.8 (18.7-25.3) | 7.92 (6.41-9.85) | 15.5 (12.5-19.3) | -1.27 (-1.44--1.11) |  |
| High-income North America | 143.11 (113.27-179.2) | 44.1 (34.6-55.5) | 266.27 (220.86-319.15) | 49.6 (40.2-60.6) | 0.54 (0.34-0.74) |  |
| Southern Latin America | 7.99 (6.78-9.51) | 17 (14.4-20.2) | 10.42 (8.33-13.03) | 13 (10.3-16.4) | -0.68 (-0.8--0.57) |  |
| Tropical Latin America | 19.42 (18.41-20.79) | 16.7 (15.9-17.9) | 43.91 (40.42-47.69) | 17.2 (15.9-18.6) | 0.11 (-0.13-0.34) |  |
| North Africa and Middle East | 29.88 (22.46-40.99) | 12.5 (9.9-16.6) | 65.23 (52.16-81.44) | 11.8 (9.5-14.6) | -0.09 (-0.13--0.05) |  |
| Central Sub-Saharan Africa | 4.27 (3-5.69) | 13 (9.3-17.1) | 10.68 (7.39-14.92) | 12.6 (9-17.3) | -0.08 (-0.16-0) |  |
| Eastern Sub-Saharan Africa | 14.15 (10.73-17.91) | 13.2 (9.7-16.6) | 33.54 (23.24-43.66) | 13.1 (9.1-17.3) | -0.07 (-0.14--0.01) |  |
| Southern Sub-Saharan Africa | 4.97 (3.91-5.97) | 13.1 (10-16.6) | 8.96 (7.4-10.63) | 13.1 (10.9-15.5) | 0.15 (-0.2-0.49) |  |
| Western Sub-Saharan Africa | 37.8 (25.9-47.1) | 28.8 (20-36.9) | 104.49 (64.78-142.65) | 30.2 (19.8-40.7) | 0.18 (0.13-0.22) |  |

IBD, inflammatory bowel disease; ASPR, age-standardized prevalence rate; UI, uncertainty interval; EAPC, estimated annual percentage change; UI, uncertainty interval.
